# Pro-Tumoral Functions of Autophagy Receptors in the Modulation of Cancer Progression

**DOI:** 10.3389/fonc.2020.619727

**Published:** 2021-02-01

**Authors:** Cristóbal Cerda-Troncoso, Manuel Varas-Godoy, Patricia V. Burgos

**Affiliations:** ^1^Centro de Biología Celular y Biomedicina (CEBICEM), Facultad de Medicina y Ciencia, Universidad San Sebastián, Santiago, Chile; ^2^Centro de Envejecimiento y Regeneración (CARE-UC), Facultad de Ciencias Biológicas, Pontificia Universidad Católica de Chile, Santiago, Chile

**Keywords:** autophagy, autophagy receptors, cancer progression, metastasis, aggressiveness

## Abstract

Cancer progression involves a variety of pro-tumorigenic biological processes including cell proliferation, migration, invasion, and survival. A cellular pathway implicated in these pro-tumorigenic processes is autophagy, a catabolic route used for recycling of cytoplasmic components to generate macromolecular building blocks and energy, under stress conditions, to remove damaged cellular constituents to adapt to changing nutrient conditions and to maintain cellular homeostasis. During autophagy, cells form a double-membrane sequestering a compartment termed the phagophore, which matures into an autophagosome. Following fusion with the lysosome, the cargo is degraded inside the autolysosomes and the resulting macromolecules released back into the cytosol for reuse. Cancer cells use this recycling system during cancer progression, however the key autophagy players involved in this disease is unclear. Accumulative evidences show that autophagy receptors, crucial players for selective autophagy, are overexpressed during cancer progression, yet the mechanisms whereby pro-tumorigenic biological processes are modulated by these receptors remains unknown. In this review, we summarized the most important findings related with the pro-tumorigenic role of autophagy receptors p62/SQSTM1, NBR1, NDP52, and OPTN in cancer progression. In addition, we showed the most relevant cargos degraded by these receptors that have been shown to function as critical regulators of pro-tumorigenic processes. Finally, we discussed the role of autophagy receptors in the context of the cellular pathways implicated in this disease, such as growth factors signaling, oxidative stress response and apoptosis. In summary, we highlight that autophagy receptors should be considered important players of cancer progression, which could offer a niche for the development of novel diagnosis and cancer treatment strategies.

## Introduction to Phases of Cancer Development

According with the World Health Organization (WHO) in 2018 around 18.1 million people in the word had cancer, and 9.6 million died due to this disease, making it the second leading cause of death worldwide (1).

**Figure 1:**
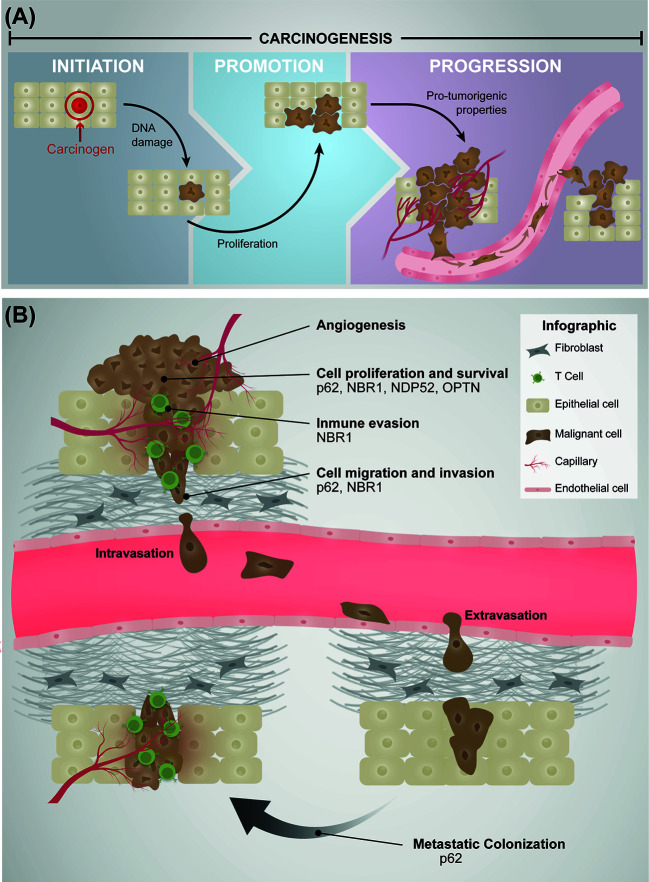
**(A)** Stages of Carcinogenesis. Initiation involves irreversibly alterations of particular tissue cells and increased susceptibility to tumor progression. The alterations are frequently related with mutational events induced with chemicals, radiation or biological agents (carcinogen). Promotion implicates the clonal expansion of altered cells leading to a visible tumor, a stage known to be reversible. In the progression stage, cells show several characteristic processes necessary to develop a malignant phenotype characterized by aggressive properties such as angiogenesis, cell proliferation and survival, immune evasion, cell migration and invasion, and metastasis. **(B)** Involvement of autophagy receptors in cancer progression. During the stage of cancer progression several characteristic processes occur. Angiogenesis corresponds to the formation of interconnected capillaries within the tumor. This is the product of the up-regulation and secretion of pro-angiogenic factors by cancer cells, a crucial process in the supply of oxygen and nutrients to the tumor. Cytosolic autophagy receptors do not promote this process. Cell proliferation and survival are the consequence of genetic changes which promotes metabolic and morphological features that sustain these events. P62, NBR1, NDP52, and OPTN are involved in the promotion of these processes by several mechanisms. Immune evasion corresponds to the mechanism by which cancer cells evaded the immune system, here represented by T cells. NBR1 is known to contribute to immune evasion. Migration and invasion processes are part of the metastatic cascade, in which cells acquire the capacity to migrate and invade the surrounding tissue of the primary tumor. Furthermore, it is proceeded by the intravasation into the circulatory or lymphatic system. p62 and NBR1 receptors promote migration and invasion processes. After intravasation, survival cells in circulation proceed to extravasation in a distant site (respect to primary tumor), and colonize and grow in a new site (metastasis colonization). p62 supports the metastatic process.

The development of cancer, termed carcinogenesis is a multistep process involving three different stages: initiation, promotion, and progression (2, 3) (**Figure 1A**). The tumor initiation and promotion involve irreversible genetic alterations in normal cells, induced by a carcinogen, followed by a reversible process regulated by epigenetic modifications, which promotes the clonal expansion of the altered cells (2) (**Figure 1A**). The final result of these two steps is the generation of a pre-neoplastic lesion forming a visible tumor (2, 4, 5). Although both, initiation and promotion are two crucial steps in cancer development, it is not until the tumor progression step is triggered that altered cells begins to express a malignant phenotype and acquire more aggressive characteristics forming cancer cells (2, 5, 6) (**Figure 1A**). In this early stage of the tumor progression, cancer cells show an increase in the frequency of additional genetic abnormalities such as number of chromosomes, single point mutations, translocations, deletions, and amplifications of genes namely *TP53*, *RB1*, *EGFR*, and *KRAS*, among others (7), which are responsible for promoting metabolic and morphological changes that sustain the proliferation of cancer cells (2, 4, 5, 8) (**Figure 1B**). In addition, to sustain the ability to proliferate, cancer cells must acquire several properties to contribute to the tumor progression, including resistance to cell death, induction of angiogenesis, evasion of the immune system, and activation of the metastatic cascade (9) (**Figure 1B**). During tumor progression, cancer cells are exposed to extreme conditions characteristic of the tumor microenvironment, however how tumor cells adapt to these adverse scenarios is only partially understood. For example, it has been shown that hypoxia, a physiological feature present in the tumor microenvironment triggers apoptosis, dependent of the tumor suppressor p53 (10). Strikingly, the *TP53* gene is frequently found mutated in cancer cells, which elicits a loss of function that ultimately results in apoptosis resistance (11). Cancer cells must also survive to the attack of immune cells, a process known as immune evasion (9) (**Figure 1B**). Here, cancer cells can evade the immune system losing the expression of MHC-I, activating either the intrinsic signaling pathway WNT/β-Catenin axis or promoting the secretion of the factor like VEGF-A, among others (12). In fact, hypoxia up-regulates the expression and secretion of VEGF-A triggering the formation of interconnected capillaries within the tumor (13). This process is called angiogenesis, which is responsible of oxygen and nutrients supply to the growing tumor, allowing cancer cells to survive and proliferate (**Figure 1B**). Angiogenesis is also required to provide an escape route to cancer cells for dissemination and colonization in distant organs through the process of metastases (14). The metastatic cascade involves the capacity of cancer cells present in the primary tumor to migrate and invade the surrounding tissues leading to intravasation in the circulatory or lymphatic system (**Figure 1B**). Cancer cells survive in the circulation, including extravasation in a distant site, with the capacity to colonize and grow in the new site (15–17).

Several cellular and signaling pathways are involved in how pro-tumorigenic properties in cancer cells are triggered, impacting multiple steps in the cascade of tumor progression (9, 18). One cellular pathway originally implicated as a tumor-suppression mechanism is autophagy, which is now considered a potent tumor promoter cellular pathway (19).

## Autophagy: A Crucial Cellular Pathway During Cancer Progression

Although several studies have shown that basal levels of autophagy can suppress initiation of tumor development (20), a growing number of studies indicated that autophagy enables tumor cell survival, growth, and malignancy by facilitating the supply of metabolic demands during tumor progression (21, 22). In fact, defects in the autophagic machinery often restrain the proliferation, dissemination, and metastatic potential of malignant cells. Indeed, pharmacological interruption of autophagy or genetic knockdown of crucial ATG proteins promoted apoptosis of tumor cells (23–27). In addition, autophagy-deficient tumors are often more sensitive to several chemotherapeutic agents as well as to radiation therapy than their autophagy-proficient counterparts (20, 28, 29). In this review, we summarize the contribution of autophagy cytosolic receptors during the tumor progression stage in carcinogenesis.

**Figure 2:**
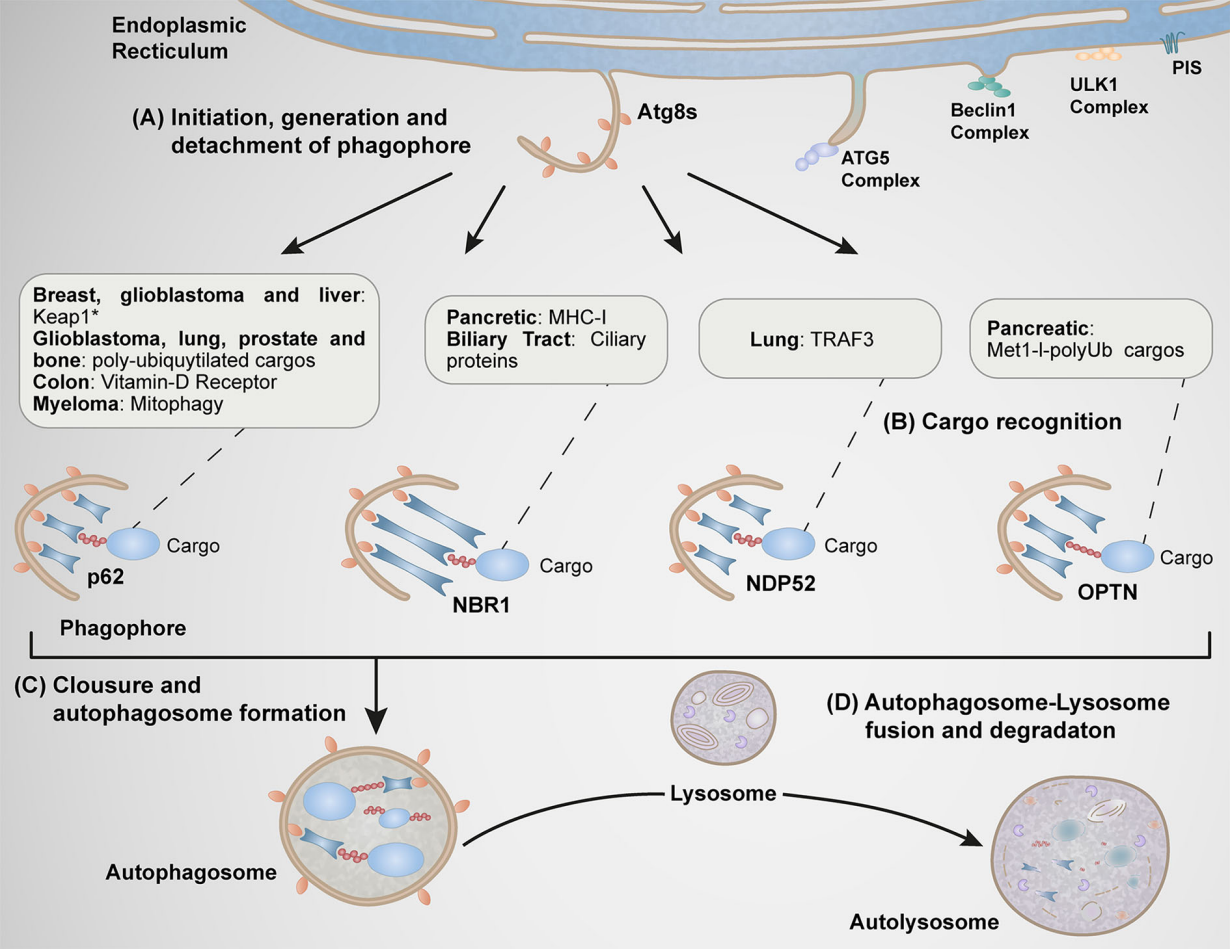
Function of autophagy receptors in different types of cancer. **(A)** At specific subdomains of the endoplasmic reticulum (ER) enriched of phosphatidylinositol synthase (PIS), various Atgs complexes are recruited (ULK1 and Beclin1 complexes). These steps are implicated during early stages of autophagosome formation. Subsequently, the Atg5 complex is recruited to this location facilitating the conversion [from a soluble cytosolic form to a phophatidylethanolamine (PE)-conjugated membrane-bound form] of Atg8 family members (LC3A, LC3B, LC3C, GABARAP, GABARPL1, and GABARAPL2), process implicated in the elongation of the membrane, structure known as phagophore. **(B)** The phagophore is further detached at the ER, where ATG8s proteins begin to interact with autophagy receptors responsible of the selective capture of cargos. **(C)** The final closure of the phagophore form a vesicular double membrane structure called autophagosome. **(D)** The autophagosome finally fuses with the lysosome forming the autolysosome. p62, NBR1, NDP52, and OPTN function in the progression stage of carcinogenesis in several types of cancer by the selective capture of specific cargos indicated in the boxes.

Macroautophagy (herein referred to as autophagy) is a catabolic process involving the engulfment of cytoplasmic material into double-membraned autophagosomes that subsequently fused with lysosomes to form autolysosomes, where the materials are finally degraded by lysosomal hydrolytic enzymes (30, 31) (**Figure 2**). Autophagy substrates included abnormal constituents such as protein aggregates, damaged organelles and intracellular pathogens (32). Autophagy is also involved in the degradation of normal cellular constituents for cell survival under restriction of nutrients or by the actions of stressors, a response necessary to maintain cellular fitness in response to environmental conditions contributing to the pathogenesis of various disorders, including cancer (30, 33).

The mechanism of autophagy consists of multiple steps, including formation and expansion of the pre-autophagosomal isolation membrane (phagophore) induced by cellular signals, substrate engulfment, autophagosome closure, and autophagosome-lysosome fusion (30, 34) (**Figure 2**).

**Figure 3:**
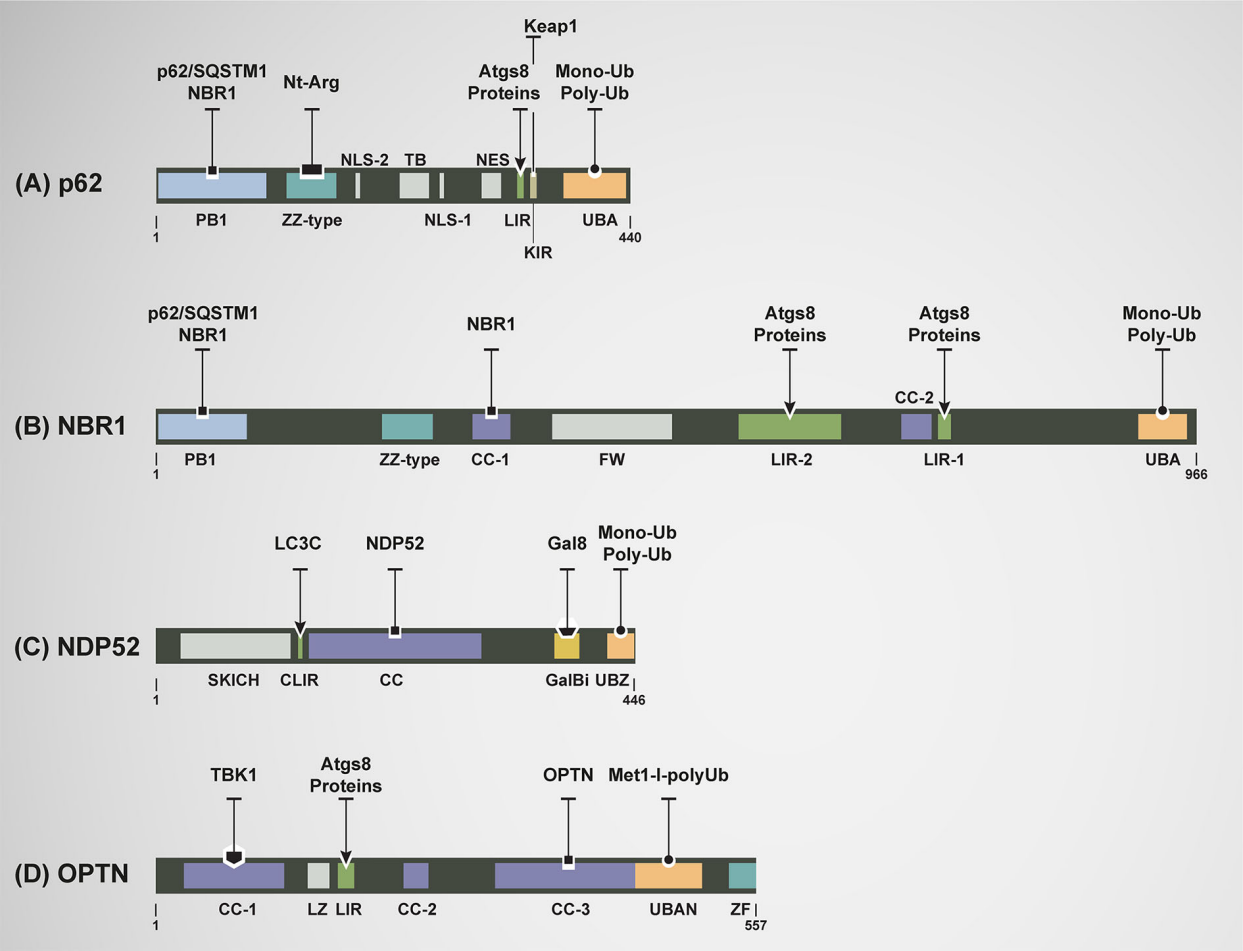
Domain architecture of mammalian autophagy receptors and relevant interactions. **(A)** p62: N-terminal region Phox-BEM1 domain (PB1) mediates p62 homodimerization or its heterodimerization with NBR1; ZZ-type zinc finger domain recognizes N-terminal argenylated substrates (Nt-Arg); nuclear localization signals (NLS1 and NLS2); tumor necrosis factor (TNF) associated receptor-6 (TRAF6) binding (TB) domain; export motif (NES); LC3-interacting region (LIR) motifs mediate the interaction with all Atg8s; KEAP1-interacting region (KIR) binding with KEAP1; and ubiquitin-associated (UBA) domain recognizes mono and poly-ubiquitylated (Mono-Ub and Poly-Ub) substrates. **(B)** NBR1: PB1 mediates interaction with p62 and itself; ZZ-type Zink finger, Coiled-coil-1 (CC-1) mediates self-oligomerization, four tryptophan (FW); LIR-2 motif; CC-2 domain; LIR-1 motif, binding to Atgs8 proteins more functional that LIR-2; and UBA domain recognizes mono-Ub and poly-Ub substrates. **(C)** NDP52: skeletal muscle and kidney-enriched inositol phosphatase carboxyl homology domain (SKICH); LC3C-specific LC3-interacting region (CLIR) mediates selective and strong binding to LC3C; Coiled-coil (CC) domain participates in its homodimerization; Galectin-8 binding region (GalBi) mediates the interaction to Galectin-8 in the context xenophay and lysophagy; and ubiquitin-binding zinc finger (UBZ) domain binds to mono-Ub or poly-Ub. **(D)** OPTN: three Coiled-coil domains are found (CC-1, CC-2, and CC-3). CC-1 domain promotes the formation of a hetero-tetramer complex between OPTN and serine/threonine TANK-binding kinase 1 (TBK1). CC-3 domain mediates the homodimerization of OPTN; a leucine zipper (LZ) domain; LIR motif binds to all members of Atg8s family; ubiquitin-binding domain of ABIN proteins and NEMO (UBAN) binds to methionine1 (Met1)-linked linear polyubiquitin (Met1-l-polyUb) of ubiquitylated cargos; and zinc finger (ZF) domain.

Cellular signals promote the formation of the phagophore at specific subdomains of the endoplasmic reticulum (ER) enriched of phosphatidylinositol synthase (35). Within these domains occurs the recruitment of several ATG proteins necessary for early events of phagophore formation and expansion that mediates the synthesis of phosphatidyl-inositol-3-phosphate (PI3P), a pivotal phospholipid needed in the later recruitment of other ATG proteins (34) (**Figure 3**). Key ATGs are members of the yeast Atg8 family of ubiquitin (Ub)-like proteins (LC3A, LC3B, LC3C, GABARAP, GABARPL1, and GABARAPL2 in mammals) which play roles in autophagosome formation and autophagosome-lysosome fusion (36, 37). The best studied member of this family is LC3B (product of the MAP1LC3B), which undergoes conversion from a soluble, cytosolic form (LC3B-I) to a phophatidylethanolamine (PE)-conjugated, membrane-bound form (LC3B-II) (38). LC3B-II subsequently interacts with LC3-interacting region (LIR) motifs of various cargo receptors to capture autophagic cargos into forming autophagosomes (39, 40) (**Figures 2**, **3**).

Selective autophagy is orchestrated by cargo receptors responsible for the recognition and incorporation of cargos into the autophagosomes (41). Among these receptors are cytosolic proteins such as p62/SQSTM1, NBR1, OPTN, NDP52, TAX1BP1, and TOLLIP. These receptors bind polyubiquitinated cargos *via* their Ub-binding domains (42, 43). Other cargo receptors are anchored to the autophagic cargos *via* their transmembrane domains, as is the case for BNIP3, NIX and FUNDC1 in mitochondrial autophagy (mitophagy) (39, 40), and RTN3, SEC62, CCPG1, FAM134B and TEX264 in ER autophagy (ER-phagy) (40, 44–48). After fusion of autophagosomes with lysosomes, the autophagy cargos, together with the Atg8-family proteins and cargo receptors, are degraded in autolysosomes (49, 50).

Interestingly, several cytosolic autophagy receptors such as p62/SQSTM1, NBR1, NDP52 and OPTN have been reported to be overexpressed in several types of cancer playing regulatory roles in the last stage of carcinogenesis (**Figures 1**, **2**). In this review, we focus on the emerging roles of autophagy receptors in cancer cell biology.

## Molecular Features of Autophagy Cytosolic Receptors

Most of cytosolic autophagy receptors are characterized by the presence of specific domains that define their role as cytosolic sensors of damaged cellular constituents. Generally, they harbor both LC3-interactin region (LIR) and ubiquitin-binding domains (UBDs) (51). The LIR motif is considered a hallmark of these receptors corresponding to a short sequence of 6 amino acids based on the multiple alignments of LIR sequences. This sequence is known to be responsible for the interaction with ubiquitin-like proteins like the lipidated ATG8-proteins (LC3s and GABARAPs) in the phagophore membrane (39, 40, 52). UBDs are modular elements found in each autophagy receptor that bind non-covalently to the protein modifier ubiquitin (39, 51). The preferences of UBDs for ubiquitin chains of specific length and linkage are central to their functions in the recognition of cargos into the autophagosomes. Most UBDs use α-helical structures to bind a hydrophobic patch in the β-sheet of ubiquitin (53). For instance, the ubiquitin-binding zinc finger (UBZ) binds ubiquitin with a single α-helix oriented either parallel or antiparallel to the central β-strand. However, other ubiquitin-binding elements, including the ubiquitin-associated (UBA) domain and ubiquitin binding in ABIN and NEMO (UBAN) domain bind ubiquitin through two discontinuous α-helices (53). Interestingly, a variety of post-translational modifications including acetylation, phosphorylation or ubiquitylation can positively regulated the LIRs and UBDs domains enhancing their affinity to ATG8s proteins and ubiquitin, respectively (54–57).

### p62/SQSTM1/Sequestosome-1 (Hereafter Referred to as p62)

p62 was the first autophagy receptor identified in mammals (50). This autophagy receptor is a multidomain protein, which contains a LIR motif that interacts with LC3s/GABARAPs attached to the autophagosomes and an UBA domain located in its C-terminal region allowing p62 to associate with ubiquitin and ubiquitin-tagged cargos. This binding results in the formation of cytosolic aggregates and/or the incorporation of cargos into autophagosomes having a functional role in cell survival (58, 59). In addition, p62 contains other additional modules with a role in autophagy such as the ZZ type zinc finger domain. This domain binds to cytosolic cargos bearing amino terminal arginine residues (Nt-Arg) generated by proteolytic processing (N-degrons), interaction that drives these cargos for autophagy degradation (60–63); and a KEAP1-interacting region (KIR) implicated in the sequestration of KEAP1, a key adaptor protein for Cullin-3 ubiquitin ligase implicated in the ubiquitylation and inactivation of the transcription factor NRF2 by degradation through the ubiquitin proteasome system (UPS) (59, 64) (**Figure 3**). Thus, p62 competitively binds to KEAP1 to allow NRF2 function, a transcription factor engaged in the control of ROS levels (65). p62 is expressed in all tissues and has been extensively studied as a scaffold protein in several signal transduction pathways, many of which have been involved in cell survival and cell death (65–68).

#### p62 and Poor Prognosis in Cancer Patients

p62 has been found overexpressed in different types of tumors, which expression has been associated with poor prognosis in cancer. For example, studies performed in patients with non-small cell lung cancer, including those with lung adenocarcinoma, showed an increase in the levels of p62, correlating with poor prognosis in this type of cancer (69). In addition, immunohistochemistry analysis of tumors derived from patients with non-small cell lung cancer demonstrated an association between high expression of p62 and the aggressiveness of the tumor (70). A similar correlation was also reported in patients with colorectal cancer, osteosarcoma, prostate cancer, hepatocellular carcinoma, breast cancer, and acute myeloid leukemia, among others (71–75).

#### p62 and Pro-Tumorigenic Properties

The association of p62 expression with the aggressiveness of several types of cancer has been investigated in different cellular models, in which the contribution of p62 in the induction of different pro-tumorigenic properties has been proven. In a cell line of lung adenocarcinoma, silencing of p62 promotes the formation of aberrant autophagosomes, condition that triggers cancer cell death (76). In the same context, reduction in the levels of p62 in a model of chemoresistance of small-cell lung cancer increases its sensitivity against cisplatin. Contrary, overexpression of p62 enhances the resistance to this chemotherapeutic agent, preventing cell death in response to this treatment (77). In the colorectal cancer cell line SW480, p62 proteins levels are found elevated (78), which correlates with active autophagy pathway compared to other cellular models of this type of cancer (79). Interestingly, silencing of p62 in SW480 cell line decreases cell proliferation and their capacity to invade and migrate. Additionally, injection of p62 depleted SW480 cells in mice decreases tumor growth and metastasis into the lung, compared to control cells (78). Similar findings have been reported with F5M2 and F4 cell lines of osteosarcoma, which present high levels of p62 (73). Silencing of p62 in these cell types decreases their proliferative capacity, migration, and invasion (73). Another example is the cell line Huh-1, a model of human hepatocellular carcinoma. Huh-1 cells present higher levels of p62 compared to the immortalized HEK293 cell line. In addition, in Huh-1 cells, p62 is found phosphorylated on its Ser349. Either silencing of p62 or expression of its ﻿phosphorylation-defective mutant Ser349A, caused a decrease in cell proliferation *in vitro*, and a reduction of tumor growth *in vivo* (65). All these studies provide strong evidence to support that p62 promotes pro-tumorigenic properties, making now necessary to elucidate how p62 mechanistically promotes tumor progression.

#### p62 and KEAP1–NRF2 Axis

One protein positively regulated by p62 is the transcription factor NRF2. As previously mentioned, p62 facilitates KEAP1 degradation, which abolishes ubiquitylation and degradation of NRF2 (80). Thus, high levels of KEAP1 upon silencing of p62, triggers a reduction in NRF2 levels. NRF2 is consider a master regulator of the cellular antioxidant response, which regulates key target genes for cancer development and progression, such as those involved in survival, proliferation, DNA repair, and autophagy (81, 82). In particular, several evidence show the contribution of NRF2 in the properties of cancer stem cells (83–85), a subpopulation of cells present in the tumor niche involved in tumor growth, therapy resistance and metastasis (86). Ryoo and colleagues showed that high levels of NRF2 are involved in drug resistance, cell migration and invasion capacity of breast cancer stem cells (87). Importantly, silencing of p62 reduces NRF2 levels, demonstrating the regulatory role of p62 on NRF2 levels in these type of cells (87). This p62/NRF2 regulation has been also found in the glioblastoma multiforme cell line T98G, which express high levels of p62. Activation of autophagy in T98G cells leads to an increase in the levels of NRF2 (88). Moreover, and similar to what occurs in Huh-1 cells, p62 in T98G is found phosphorylated in Ser349 (88). Authors found this post-translational modification increases affinity of p62 to KEAP1 promoting its degradation by selective autophagy, with a positive impact in the stability and function of NRF2 (65). Finally, same findings have been reported in the cellular model of prostate cancer DU145, characterized by high levels of p62. Indeed, silencing of p62 in DU145 cells decrease cell proliferation, apoptosis-resistance and invasion by a mechanism related with the inactivation of the NRF2 pathway (75, 89). Altogether, these findings demonstrate the role of the axis p62/NRF2 in tumor progression in different types of cancer.

#### p62 and Other Possible Targets

p62 regulates several other proteins involved in tumor progression. One interesting target is the Vitamin D receptor (VDR). The VDR has a protective role in cancer due to its anti-proliferative and pro-apoptotic actions (90). In fact, VDR downregulation is associated with a poor prognosis and cancer progression (91). In this regard, high levels of p62 are correlated with a decrease in the levels of VDR in colorectal cancer (78), probably mediated by selective autophagy degradation. The authors showed that through its direct interaction, p62/VDR contributed to the pro-tumorigenic properties of two cell lines of colorectal cancer (SW480 and HCT116), promoting tumor progression *in vivo* (78). Another target of p62 is the transcription factor TWIST1, a crucial protein that facilitates epithelial mesenchymal transition (EMT) (92). Interestingly, the ubiquitin-associated domain of p62 interacts with TWIST1 to block its degradation by autophagy (92). Strikingly, overexpression of p62 in the A431 human skin cancer cell, which does not express TWIST1, is unable to increase cell migration. In contrast, when p62 is overexpressed together with TWIST1 in A431 cells, an increase in cell migration, tumor growth and metastasis is observed, proposing a functional link between p62/TWIST1 in promoting pro-tumorigenic effects *in vivo* (92). Another protein implicated in the pro-tumorigenic effects of p62 is Vimentin, a protein involved in tumor progression. Vimentin is a Type III intermediate filament that regulates cell shape, motility, and adhesion during EMT, processes implicated in cell invasion and aggressiveness in cancer cells (93, 94). In the highly metastatic MDA-MB-231 breast cancer cell line, vimentin co-immunoprepicitates with endogenous p62. Interestingly, silencing of p62 leads to a decrease in the levels of Vimentin correlating with a reduction in the invasive capacity of these cells. Importantly, overexpression of Vimentin is sufficient to rescue this tumoral property (71). Besides, the increase phenotype in invasive capacity of MDA-MB-231 by overexpression of p62 is dependent on Vimentin levels, demonstrating Vimentin plays a crucial role in p62-mediated invasion in breast cancer cells (71). The molecular mechanism by which p62 regulates Vimentin levels remains unknown. However, and similar to the findings with TWIST1, it is possible that p62 could act by preventing Vimentin degradation. In addition, p62 is also implicated in the selective degradation of dysfunctional mitochondrial by mitophagy in acute myeloid leukemia cells (74). In fact, p62 promotes myeloid transformation, cell proliferation, leukemia development and progression of acute myeloid leukemia by a process dependent on the efficient degradation of mitochondria by mitophagy (74).

### Neighbor of BRACA1 Gene1

NBR1 (neighbor of BRCA1 gene1) is an autophagy receptor with several domains including a PB1, CC1, LIRs, and UBA (95). Its PB1 domain, allows NBR1 oligomerization with either itself or p62 where these two receptors act either independently or cooperatively in the recognition of cargos for degradation (95). Similar to PB1, CC1 domain also facilitates NBR1 oligomerization. Indeed, deletion of the CC1 domain on NBR1 impairs its oligomerization and avidity to bind ubiquitin (96). Both LIRs domains can individually interact with Atg8s-proteins, where LIR1 is the most functional domain (95). Finally, the UBA domain mediates binding of NBR1 to monoubiquitin or poly-ubiquitin chains (51, 96) (**Figure 3**). Although the most common function of NBR1 is associated with its role as an autophagy receptor of autophagosomes, NBR1 can also be found associated with endosomal membranes, where it seems to mediate the delivery of certain cargos (52, 96–98). In terms of expression, NBR1 is expressed in all tissues, showing its highest expression in testis and thyroid (99).

#### NBR1 in Cancer Patients

Little information is known about the role of NBR1 in cancer. Data extracted from the human protein atlas (www.proteinatlas.org) showed mRNA expression of NBR1 in 17 different types of cancer with low cancer specificity (**Supplementary Figure 1**), whereas NBR1 protein levels in cancer samples displayed weak to moderate cytoplasmic expression (100). Related to cancer prognosis, it has been reported that low mRNA levels of NBR1 predict a poor clinical outcome in patients with clear cell renal carcinoma (101).

#### NBR1 in Migration and Metastasis

Although the clinical data available show a negative association between the NBR1 mRNA levels and the prognosis of patients with cancer, other findings suggest a positive contribution of NBR1 in the acquisition of pro-tumorigenic properties. For example, it is known that NBR1 contributes for cancer cell migration, a process finely regulated by structures called focal adhesions (FAs), a large protein complex that connects tumor cells with the extracellular matrix (ECM) through the action of integrins (102). Turnover of the FAs is essential for the migratory rate of tumor cells dependent on the assembly and disassembly of these complexes, processes that impact positively pro-tumorigenic properties (103). For instance, in a cellular model of breast cancer cell known as HRAS-transformed MCF10A cells that mimic an early stage of the tumor progression cascade, NBR1 binds ubiquitylated proteins of FAs mediating their degradation by autophagy. Indeed, reduction of NBR1 levels reduces FAs turnover with a negative impact in breast cancer cell migration (104). This effect is not observed in other breast cancer cell models, indicating some level of specificity of NBR1 function depending on the cell type and stages of the tumor progression. Accordingly, recent studies have demonstrated that NBR1 plays an important role in breast cancer metastatic progression. First, it was demonstrated that autophagy promotes growth of the primary breast cancer tumor but with a negative impact in the metastasis stage. In contrast, inhibition of autophagy showed an impairment in tumor growth but with a positive impact in metastasis (105). Moreover, it was found a robust accumulation of NBR1, suggesting that intracellular accumulation of NBR1 plays a role on metastasis (105). Interestingly, ectopic NBR1 overexpression in breast cancer cells is sufficient to promote metastatic outgrowth. Contrary, silencing of NBR1 suppresses cancer dissemination. However, the mechanism by which NBR1 promotes metastasis is still unknown. Since the effect of NBR1 on metastasis is related with inhibition of autophagy, it opens the possibility that NBR1 mediates metastasis by a non-canonical function possibly related with its role on endosome membranes. In this regard, it has been shown that NBR1 ﻿prevents the degradation of tyrosine kinase receptors, such as epidermal growth factor receptor (EGFR) and fibroblast growth factor receptor (FGFR), causing the accumulation of these cargos in endosome compartments (98), a key aspect in the control of their signaling (106–111).

#### NBR1 Function in Evasion of the Immune System

The immune system has the potential to recognize and eliminate tumor cells, therefore escape to the immune surveillance, which contributes to cancer progression (12). ﻿A common mechanism used by tumor cells to evade the immune system, specifically CD8+ T cells, is the impairment of the antigen presentation, which can be the result of mutations or loss of the expression of the major histocompatibility complex class I (MHC-I) (112–114). ﻿In pancreatic ductal adenocarcinoma, resistant mostly to all therapies, MHC-I is found downregulated due to the consequence of mutations in MHC-I (114). Furthermore, MHC-I is not found at the cell surface of these cells, instead it accumulates in intracellular membranes. Surprisingly, silencing of ULK1/2 complex (ULK1, FIP200 or ATG13), a protein complex implicated in the initiation of autophagosome biogenesis is sufficient to rescue the levels of MHC-I at the cell surface (114). ﻿Among all autophagy receptors, it is known that NBR1 interacts with ubiquitylated MHC-I. Moreover, silencing of ﻿NBR1 rescues the levels of MHC-I at the cell surface of pancreatic ductal adenocarcinoma cells. Together, all these antecedents strongly indicate that distribution of MHC-I at the cell surface is controlled by NBR1 selective autophagy, highlighting NBR1 as a crucial molecule in how tumor cells evade the immune system (114).

#### NBR1 and Loss of Primary Cilium in Cancer

Primary cilia are non-motile microtubule-based cellular organelles present in nearly every cell that gather information about the environment, triggering a variety of cellular responses through specific intracellular signaling pathways (115). The primary cilium is dynamically regulated during the cell cycle, disappearing transitorily during cellular division (116). Importantly, loss of primary cilia has been reported in different cancer cells and tumoral tissues including pancreatic, renal, and hepatic carcinomas (117, 118). Interestingly, it has been reported that in a cellular model of cholangiocarcinoma, a type of hepatic cancer, autophagosomes are located in the primary cilia, suggesting a role of autophagy in their maintenance. Indeed, LC3 interacts with the ciliary proteins IFT88 and α-tubulin. Moreover, in comparison with others autophagy genes, NBR1 expression is found increased in intrahepatic cholangiocarcinoma tumor samples compared to normal controls. In addition, silencing of NBR1 in HuCCT1 cells, a cell line of cholangiocarcinoma, increases the size of the primary cilia (119). These antecedents suggest that NBR1 could be implicated in the degradation of ciliary component through selective autophagy, explaining the loss of the primary cilium in cholangiocarcinoma.

### Nuclear Dot Protein 52 KDa

Nuclear dot protein 52 KDa [NDP52, also known as calcium binding and coiled-coil domain 2 (CALCOCO2)] is composed by the skeletal muscle and kidney-enriched inositol phosphatase carboxyl homology domain (SKICH), LC3C-specific LC3-interacting region (CLIR), Coiled-coil (CC), Galectin-8 binding region (GalBi), and ubiquitin-binding zinc finger (UBZ) domain (51, 120, 121). In mammals, *NDP52* is located on the chromosome 17 and is composed of 15 exons. The role of SKICH domain in autophagy is not yet completely understood. However, it is known that SKICH domain is responsible in the binding of NDP52 to the mitochondrial RNA poly(A) polymerase (MTPAP) in depolarized mitochondria to enhance mitophagy (122). On another hand, the CLIR domain in NDP52 is a non-canonical LIR motif that confers selective and strong binding to LC3C, with a very weak affinity to other Atg8s proteins members (120). The CC domain in NDP52 participates in its homodimerization facilitating the binding to LC3C (121, 123). The GalBi domain allows the binding of NDP52 to Galectin-8 in the context of degradation of pathogens (xenophagy) or damaged lysosomes (lysophagy), selective forms of autophagy (124, 125). Finally, the UBZ domain allows NDP52 binding to ubiquitin (mono or poly-ubiquitin) (51, 58) (**Figure 3**).

#### NDP52 and Its Role in Cancer Cell Survival

NDP52 has been detected in different cancer tissues with a moderated protein expression, including the majority of renal cancers. In contrast, in few cases of malignant gliomas, malignant lymphomas, skin, and lung cancers, NDP52 protein expression is almost undetected [Human protein atlas (99)]. Although the role of NDP52 in cancer is still unknown, recent evidence suggests that NDP52 could have a role in the acquisition of some pro-tumorigenic properties such as cell survival. For instance, in the cellular model of non-small cell lung cancer, cell line A549, NDP52 is found bound to LC3 in autophagosomes under basal conditions. NDP52 mediates selective degradation of the tumor necrosis factor receptor-associated factor 3 (TRAF3), a repressor of activation and nuclear translocation of RELB, an effector of non-canonical NF-κB signaling, which is usually implicated in pro-tumorigenic properties (126, 127). Interestingly, silencing of NDP52 impairs the localization of RELB into the nucleus and downregulated the expression of anti-apoptotic target genes of REL-B (128). In addition, activation and translocation of RELB due to the degradation of TRAF3 by NDP52, inhibits the transcription factor SMAD leading to a reduction in the expression of the transforming growth factor β (TGFβ), with known tumor-suppressive functions (126, 129). This inhibition promotes proliferation of A549 cells and tumor growth in animal models of non-small cell lung cancer (126).

### Optineurin

Optic neuropathy inducing, also called Optineurin (OPTN), is composed by three Coiled-coil domains (CC-1, CC-2, and CC-3), a leucine zipper (LZ), LIR, ubiquitin-binding domain of ABIN proteins and NEMO (UBAN) and zinc finger (ZF) domain (130). The CC-1 domain, located in the N-terminal of OPTN binds serine/threonine TANK-binding kinase 1 (TBK1) leading to the formation of a stable OPTN-TBK1 hetero-tetramer complex. TBK1 phosphorylates the Ser172 on LIR domain of OPTN enhancing its binding to ATG8s proteins. In addition, TBK1 phosphorylates the Ser473 located on the UBAN domain leading to an increase in the binding to ubiquitin (131). The LIR domain binds to all members of ATG8s family, but compared to other autophagy receptors, the LIR domain of OPTN is the unique phosphorylated by TBK1 (51, 132). The CC-3 domain mediates the homodimerization of OPTN. Only in this form, OPTN binds, through the UBAN domain, to methionine1 (Met1)-linked linear polyubiquitin (Met1-l-polyUb) of ubiquitylated cargos in a reason of 2:1 (53, 131, 133) (**Figure 3**).

#### OPTN in Cancer Tumor Progression

RNA-seq data of 17 different types of cancer show that OPTN is overexpressed in pancreatic cancer, being the second most expressed autophagy receptor, after p62, in this type of cancer, and its expression correlated with a reduced survival of pancreatic ductal adenocarcinoma patients (134). Furthermore, the silencing of OPTN in different cells lines of pancreatic ductal adenocarcinoma promotes cell cycle arrest, decreases colony formation and induces apoptosis through ER stress activation (134). These antecedents indicate OPTN could play a relevant role in pancreatic ductal adenocarcinoma cells. However, it is necessary to find new cargos, which could work as negative regulatory proteins of the cell cycle.

## Cancer Therapy and Selective Autophagy

Chemotherapy is the main strategy for cancer treatment, characterized by the use of drugs that alter and kill tumoral cells rapidly (135). These drugs include anti-mitotic agents (e.g., paclitaxel and docetaxel), topoisomerase II inhibitors (e.g., doxorubicin and epirubicin) and DNA alkylating agents (e.g., cisplatin and carboplatin) (135). Regrettably, tumor cells respond developing a variety of cellular adaptation programs that provide the ability to tolerate the cytotoxic effects of chemotherapy (135, 136). One of this responses is the activation of autophagy, a pathway that helps in the evasion of the effects of chemotherapies in tumor cells transforming them in cells resistant to chemotherapy (135–138). Indeed, it has been previously summarized the contribution of autophagy in chemoresistance in different types of tumor under different chemotherapeutic agents, proposing that autophagy inhibition is a good strategy to promote sensitization to chemotherapy (135). However, the role of autophagy receptors in chemoresistance has been poorly explored. Only recent studies have started to propose p62 as a possible target of intervention (139, 140). Cisplatin is one of the most used chemotherapeutic agent, but several studies have reported development of resistance to this chemotherapeutic agent (141). Alsamman and El-Masry showed that cisplatin promotes the increase in p62 levels in cellular models of breast, colon and ovarian cancer (139). Interestingly, treatment of these cells with cisplatin in combination with Staurosporine (natural broad-spectrum antitumor agent derived from *Streptomyces staurosporeus* (142–144) abrogates the up-regulation of p62, suggesting that Staurosporine sensitizes cancer cells to cisplatin in a p62-dependent manner (139). Similarly, Sorafenib, a multikinase inhibitor chemotherapeutic agent used in the treatment of Hepatocellular carcinoma has shown, in some cases low efficacy due to development of resistance to this chemotherapeutic agent (140). Sorafenib causes the upregulation of the KEAP1-NRF2 axis associated with an increase in the phosphorylation of p62 at Ser349 and chemoresistance (140, 145). Surprisingly, blocking interaction between KEAP1 and phospho-p62 at Ser349 seems to be sufficient to sensitize resistant cells to Sorafenib (140).

## Conclusion and Future Perspectives

Several evidences indicate that autophagy receptors play a crucial role in cancer progression. Among all autophagy receptors identified, p62 is by far the most characterized one, currently considered a good predictor marker of the grade of malignancy in several types of cancer (71–75). Since the pro-tumoral effects of p62 are not only related with the degradation of specific cargos such as what occurs with VDR or damaged mitochondria, it opens the possibility of non-canonical roles of p62 mostly related with the stability of certain proteins like NRF2, TWIST1 and Vimentin (65, 71, 75, 80, 87). A challenge for the future to better understand the contribution of p62 during cancer progression is the identification of novel cargos of this receptor, considering specific types of cancer cells including cancer stem cells. Furthermore, it is critical to investigate these aspects studying the variety of stages during cancer progression. This type of approach could offer valuable information for the design of novel strategies in cancer treatment reducing the side effects commonly observed with current treatments. In addition to p62, recent findings highlight the role of NBR1 in cancer progression, controlling the presence of important molecules and structures implicated in pro-tumorigenic properties such as MHC-I, FAs and cilia (104, 114, 119). It is now key to decipher the regulatory mechanisms underlying their specific recognition. Similarly, it opens the possibility of NBR1 functioning as a crucial regulator of cancer signaling pathways associated with EGFR and FGFR (98). Although there is very little information about the pro-tumorigenic roles of NDP52 and OPTN, its presence in several types of cancer, and even its overexpression in the case of OPTN, make these receptors interesting targets to study during tumor progression. In this regard, since OPTN and NDP52 participate in mitophagy (125, 146, 147), it is relevant to explore the contribution of active mitophagy pathway in tumor progression and metastasis.

In conclusion, autophagy receptors are interesting molecules with validated contribution in different tumoral contexts that promotes a variety of cellular properties during cancer progression, and therefore must be considered possible targets for cancer treatment.

## Author Contributions

CC-T, MV-G, and PB organized the entire manuscript, wrote the draft, and revised the last version of the manuscript. Figures were designed by CC-T. All authors contributed to the article and approved the submitted version.

## Funding

This research was funded by Fondo Nacional de Desarrollo Científico y Tecnológico of Chile (FONDECYT; http://www.conicyt.cl/fondecyt), grant numbers 1190928 (MV-G) and 1171649 (PB), by ANID/AFB170005 and by Proyecto ANILLO ACT172066.

## Conflict of Interest

The authors declare that the research was conducted in the absence of any commercial or financial relationships that could be construed as a potential conflict of interest.
