# Supplementary material for: Pro-Tumoral Functions of Autophagy Receptors in the Modulation of Cancer Progression
Source: Front Oncol. 2021 Feb 1;10:619727. doi: 10.3389/fonc.2020.619727 (PMC7902017; doi:10.3389/fonc.2020.619727)
Supplement: Supplementary file 1 [file DataSheet_1.pdf]

Supplementary Material

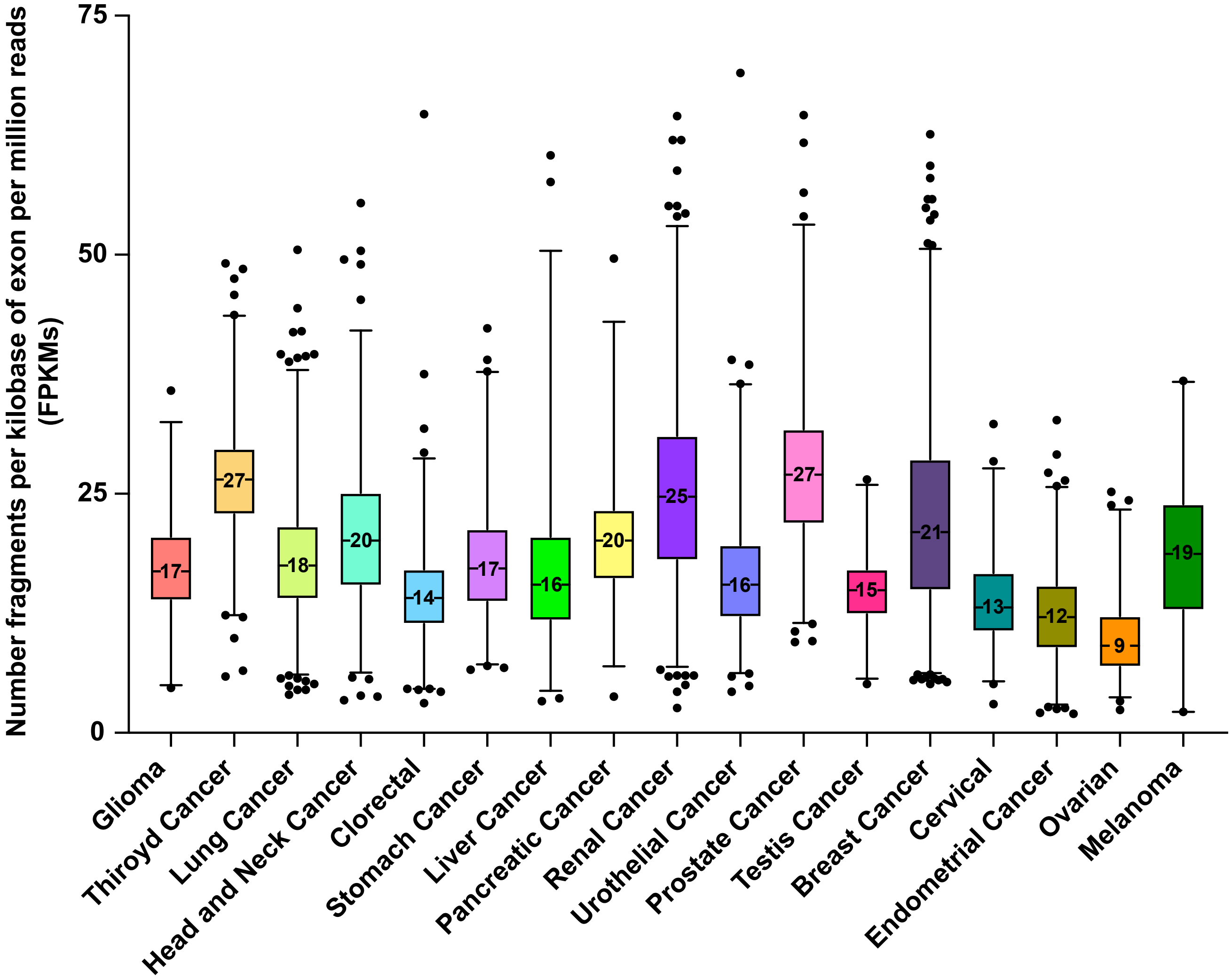

Supplementary Figure 1. BoxPlot of mRNA expression of NBR1 reported in 17 cancer types. Data were extracted from the human protein atlas (www.proteinatlas.org) and correspond to RNA-seqs reported as FPKMs (number fragments per kilobase of exon per million reads) generated by the cancer genome atlas (TCGA). According to parameters of human protein atlas platform, the levels of mRNA of NBR1 indicate that this has low cancer specificity. The number inside of the box correspond to the median and points to atypical data.
